# *Euglena gracilis* β-Glucans (1,3): Enriching Colostrum of Sow for Enhanced Piglet Immunity

**DOI:** 10.3390/ani13223490

**Published:** 2023-11-12

**Authors:** Rafael Humberto de Carvalho, Marco Aurélio Callegari, Cleandro Pazinato Dias, Susanne Kirwan, Mara Cristina Ribeiro da Costa, Caio Abércio da Silva

**Affiliations:** 1 Department of Zootechnology, Center of Agrarian Sciences, State University of Londrina, Londrina 86057970, PR, Brazil; rafael.carvalho@uel.br; 2 Akei Animal Research, Fartura 18870970, SP, Brazil; contato@akei.agr.br (M.A.C.); cleandro@cleandrodias.com.br (C.P.D.); 3 Kemin Industries, Des Moines, IA 50317, USA; susanne.kirwan@kemin.com; 4 Kemin Animal Nutrition & Health, South America, Valinhos 13279450, SP, Brazil; mara.costa@kemin.com

**Keywords:** beta-glucan, immunity, immunoglobulins, piglets, sow

## Abstract

**Simple Summary:**

Beta-glucan (βG) has been widely employed in animal diets, acting as a tool for intestinal health, improving immune status, and promoting enhanced performance. However, its origin can be from cereal grains, fungi, and algae, and in this context, its glycosidic linkages (1,3), (1,4), or (1,6) directly influence its functions in animal organisms. In general, it was observed that supplementing sow feed with 200 g of βG-(1,3) derived from the algae *Euglena gracilis* per ton, starting from the 85th day of gestation, resulted in enhancements in both colostrum production and the concentrations of immunoglobulins A, G, and M. This suggests a potential role as an immunomodulatory agent. This supplementation can become a tool to quantitatively and qualitatively enhance the colostrum provided to piglets.

**Abstract:**

The effects of supplementing the diet of sows with βG-(1,3) derived from *Euglena gracilis* algae were assessed regarding quality and amount of colostrum as well as performance of piglets. A total of 120 sows (first (nulliparous) to sixth parity (multiparous)) from D85 of gestation until weaning were divided into two groups: the control diet group (n = 60) and the βG-(1,3) diet group (n = 60). Sows receiving βG-(1,3) exhibited an average increase of 870 g (24.9%) in colostrum production, leading to a 25.17% higher intake of colostrum by piglets. Furthermore, piglets in the βG-(1,3) group showed significantly superior weight gain of 34 g (50%) compared to the control group 18 h after birth (*p* < 0.05). Sows fed with βG-(1,3) produced colostrum with significantly higher concentrations of IgG (5.914 mg/mL, 16.16%) and IgM (0.378 mg/mL, 16.29%) than the control group (*p* < 0.05). Similarly, serum concentrations of IgG (13.86 mg/mL, 51.25%), IgA (17.16 mg/mL, 120.19%), and IgM (13.23 mg/mL, 144.78%) were significantly higher in sows fed with βG-(1,3) than in the control group (*p* < 0.05). Supplementing sows with βG-(1,3) derived from the *Euglena gracilis* algae resulted in increased colostrum production and consumption, along with greater weight gain in piglets during the first 18 h after birth. Additionally, both the colostrum produced by the sows and the blood serum of the piglets exhibited higher concentrations of immunoglobulins.

## 1. Introduction

The use of probiotics, prebiotics, organic acids, and essential oils as feed additives in animal nutrition, replacing growth-promoting antibiotics, is on the rise. These additives have demonstrated the recognized ability to prevent enteric diseases and improve immune status [1], with positive repercussions on weight gain, feed intake, and feed efficiency [2,3,4].

β-glucans (βGs) are considered prebiotics and are naturally present in cereal grains, fungi, and algae [5,6,7]. They represent one of the most widely used classes of alternative additives in commercial pig production. Composed of a heterogeneous group of polysaccharides, βGs are structured with glucose molecules, linked mainly by glycosidic bonds (1,3), (1,4), or (1,6) [8].

The biological properties of βGs are recognized, and in vitro studies include antitumor effects [9] immunomodulatory effects [10,11]. These actions also extend to in vivo conditions, such as the demonstrated immune-stimulatory effect in humans [12], rats [13], and pigs [7,14]. However, not all types of βG exhibit similar immunomodulatory effects. For example, cellulose, a βG (1,4), does not hold this action [15]. These differences arise from the physical-chemical properties of βG, including purity, solubility, molecular weight, degree of branching, polymeric charge, and chemical and tertiary structure of their molecule [8].

However, βG-(1,3) derived from fungi and yeasts presents well-established benefits, acting on the regulation of the immune system in both humans and animals [7,8]. βG-(1,3) has a triple helix structure that can facilitate the interaction of molecules and cellular receptors, inducing biological effects. Due to the deficiency of β-glucanase in animals, βG-(1,3) escapes enzymatic digestion in the upper gastrointestinal tract and appears in the small intestine as a fermentation substrate for non-pathogenic microorganisms.

In this line, Kim et al. [7] evaluated βG-(1,3) derived from *Euglena gracilis* and indicated that its use as a feed supplement improved intestinal barrier function and immunity in piglets, in addition to reducing post-weaning diarrhea. Leonard et al. [16] investigated the effects of dietary supplementation with a seaweed extract (*Laminaria* spp.), a source of βG-(1,3) and (1,6), for sows and weaned piglets on post-weaning performance, intestinal morphology, intestinal microbiota, volatile fatty acid concentrations, and immune status and observed improved post-weaning performance compared to piglets weaned from unsupplemented sows. Additionally, the authors verified the reduction in *Escherichia coli* and *Enterobacteriaceae* populations in the colon and the promotion of MUC2 mRNA abundance in this organ.

However, most commercially available βGs are derived from yeasts, whereas studies involving βG-(1,3) derived from algae are scarce. Additionally, it should be considered that there are different types of algae and therefore different βG in their compositions that present distinct action potentials [17].

Derived from *Euglena gracilis*, βG contains more than 50% βG-(1,3) in its composition and has a recognized modulating function of the immune system [7,18] and proven efficiency in improving performance, especially in pigs in nursery and growing-finishing phases [14,19]. However, for pregnant and lactating sows, the effects of βG on improving body condition and reproductive performance are scarcer and less evident [20,21,22].

In this study, we aimed to evaluate the effects of dietary supplementation with βG-(1,3) derived from *Euglena gracilis* on pregnant sows from the 85th day of gestation to the end of lactation, focusing on sow reproductive performance, piglet immune profile, and diarrhea incidence.

## 2. Materials and Methods

### 2.1. Animals, Diets, and Experimental Design

The study was conducted following the recommendations of the National Council for the Control of Animal Experimentation (Conselho Nacional de Controle de Experimentação Animal—CONCEA) and was approved by the Ethics Committee on Animal Experimentation of Akei Animal Research (protocol number: 001/2021). The evaluation was carried out in a commercial farm with a capacity of 2500 sows. A total of 120 DB90 sows (DB^®^ Swine Genetics, Patos de Minas, MG, Brazil) from different parity orders (1st to 6th order) and their respective litters were evaluated during the period from the last third of gestation (D85) to complete lactation (0 to 21 days). The sows in this study were categorized by parity order: 1st (24 sows), 2nd (25 sows), 3rd (17 sows), 4th (18 sows), 5th (16 sows), and 6th (20 sows).

During the gestation phase, sows were housed in individual stalls with an area of 1.40 m^2^ and an automatic feeding system (drops). In the lactation phase, sows were housed in individual farrowing crates with an area of 6.05 m^2^, equipped with a central iron crate and a piglet’s shelter, fully slatted floor, bite ball drinker for sows, nipple drinker for piglets, and automatic feeders for sows and linear feeders for piglets. The thermal control of the piglet shelter was ensured by using electric heating. The maternity environment was climate-controlled by negative pressure.

The experimental design was randomized blocks (formed based on the parity order of the sows), with two treatments: control and βG-(1,3), with 60 repetitions per treatment, with sow and its respective litter as the experimental unit. During the final phase of gestation (85 days to 115 days of pregnancy), sows received 3.20 kg of feed/day with 640 mg/day of βG-(1,3). Throughout the lactation period, feed was provided ad libitum through an automated feeder with approximately 1200 mg/day of βG-(1,3) intake, estimated at 6 kg/day by the sows. Piglets received pre-starter feed from the 5th day of life.

The βG-(1,3) used in this study was ALETA™ (dried *Euglena gracilis* algae) provided by Kemin Industries, Inc. The product contained more than 50% β-glucan, with 100% in the form of βG-(1,3), and was provided on top of the drops for gestating sows and in the trough for lactating sows. In both evaluated periods, the product was provided in a single feed, corresponding to a dosage of 200 g/t of feed or 200 mg/kg of feed. Water was offered ad libitum throughout the experimental period. In both treatments, the diets were isoenergetic and isonutrient and free from any performance-enhancing additives. Their compositions and nutritional and energetic values according to the phases are shown in Table 1.

### 2.2. Evaluations

On the 85th day of gestation (start of the trial), on the day of farrowing, and on the 21st day of lactation, backfat thickness at point P2 and visual body score (scale of 1 to 5) were evaluated. At the end of the phase, the loss of backfat thickness during the period was calculated. The following zootechnical indices were also considered: total number of piglets born, total number of live births, stillborn and mummified piglets, average and total weight at birth, daily weight gain of piglets during the phase, litter feed conversion (sow feed consumption during the period/litter weight gain), piglet mortality rate in the maternity ward, and sow feed consumption. Dystocic parturition was defined as a situation where no piglet was delivered for over 60 min, necessitating a manual obstetric intervention. In such cases, accessible fetuses were gently assisted by manual extraction. In instances where there were no piglets in the birth canal during manual obstetric interventions and no uterine contractions were observed, the use of oxytocin during farrowing was allowed.

At the time of farrowing, a drop of blood was collected from the auricular vein with a 25 × 8 gauge needle for glucose analysis with a portable glucometer (Accu-Chek Guide Meter™, Roche Diabetes Care, Inc., Basel, Switzerland). Glucose measurement occurred at the beginning of farrowing (initial glucose), two hours after the start of farrowing (2 h glucose), and at the end of farrowing (final glucose), with the start of farrowing defined as the birth of the first piglet and the end of farrowing as the beginning of placenta expulsion after the birth of the last piglet. The duration of farrowing was also computed, defined as the interval between the birth of the first and last piglet. Sixty sows and their respective litters, thirty from each treatment, were used for these procedures.

To predict colostrum production based on piglet weight at birth and 18 h later, we utilized the equation described by Devillers et al. [23]:

CI = −217.4 + 0.217 × t + 1,861,019 × BW/t + BWb × (54.80 − 1,861,019/t) × (0.9985 − 3.7 × 10^−4^ × tFS + 6.1 × 10^−7^ × tFS^2^)

where CI = colostrum intake from t0 (g); BW = actual body weight (kg); BWb = body weight at birth (kg); t = time elapsed from first and the second weighing (min); tFS = interval between birth and first sucking (min).

Colostrum intake was calculated as a percentage of the piglets’ body weight. For the quantification of colostrum consumption, information was used from the same 60 sows and their respective litters, with 30 from each treatment. Animal weighing procedures and feed consumption calculations, minus leftovers and losses, were performed from birth to the end of lactation (21 days). Piglet weighing was performed individually at birth and weaning.

The litter diarrhea score was performed daily, as reported by Liu et al. [24], classified as: 0, normal consistency feces; 1, soft; 2, pasty; 3, watery. The litter was considered to have diarrhea when more than 20% of the litter presented this condition. The Diarrhea Index (DI) was calculated based on the following formula:

$$\mathrm{DI}=\frac{\mathrm{animals}\;\mathrm{with}\;\mathrm{diarrhea}\;\mathrm{score}\;\geq \;2\;(n)}{\mathrm{animals}\;\mathrm{per}\;\mathrm{treatment}/\mathrm{group}\;(n)}$$

Up to 6 h postpartum, a colostrum pool was obtained manually per sow, totaling 20 sows per treatment, and at 4 days postpartum, 2 mL of blood were collected from 20 randomly selected piglets, 1 from each litter. The blood was centrifuged and the serum plus colostrum were subjected to an ELISA test to determine IgA, IgG, and IgM titers (BETHYL, Pig IgG ELISA Kit, Pig IgA ELISA Kit, and Pig IgM ELISA Kit).

### 2.3. Statistical Analysis

Normal data distribution was analyzed using Kolmogorov–Smirnov and Lilliefors tests and Shapiro–Wilk’s W (*p* > 0.05), and for outlier removal, the Box and Whisker package was applied. Normal parametric data were subjected to analysis of variance and means to Student’s *t*-test. Non-normal quantitative data were compared by Wilcoxon–Mann–Whitney test and Chi-square test using Statistic for Windows^®^, version 10.0. For all tests, a *p* value equal to or less than 0.05 was considered significant and a *p* value between 0.05 and 0.10 was considered a trend.

## 3. Results

The backfat thickness values of sows on the 85th day of gestation, at the time of farrowing (D0), and at the 21st day of lactation (weaning), as well as the difference between the periods, did not differ between the analyzed groups (Table 2; *p* > 0.05). Similarly, feed conversion was not affected by the presence of βG-(1,3) in the sows’ diet (*p* > 0.05).

The addition of βG-(1,3) in the sows’ diet did not affect reproductive variables (Table 3; *p* > 0.05) except for colostrum production, colostrum intake, and piglet weight gain (Table 3; *p* < 0.05). Sows fed with βG-(1,3) increased colostrum production by an average of 870 g (24.9%) compared to sows fed with a control diet (*p* < 0.05). Consequently, piglets from these [βG-(1,3)] sows ingested 59.13 g or 25.17% more colostrum and had a higher weight gain (18 h) of 34 g (50%) compared to the control treatment. Serum glucose at different lactation times was not influenced by the addition of βG-(1,3) in the diet (*p* > 0.05).

Sows that received βG-(1,3) supplementation produced colostrum with elevated concentrations of IgG and IgM in piglets from the βG-(1,3) group (Table 4). Specifically, IgG levels were 5.914 mg/mL (16.16%) higher, and IgM concentrations were 0.378 mg/mL (16.29%) higher than those in the control group (*p* < 0.05). In this line, serum concentrations of IgG, IgA, and IgM exhibited notable increases, measuring 13.86 mg/mL (51.25%), 17.16 mg/mL (120.19%), and 13.23 mg/mL (144.78%), respectively, in comparison to the control group (*p* < 0.05).

The performance of litters (Table 5) from sows fed with βG-(1,3) on days 2 (D2), 10 (D10), 18 (D18), and 21 (D21) was not influenced compared to the control group (*p* > 0.05). The incidence, index, and total number of litters with diarrhea during the lactation period (Table 6) were not impacted by the addition of βG-(1,3) in the sows’ diet (*p* > 0.05). However, in the day-to-day analysis, on days 8 (D8) (with values of 0.136 versus 0.017) and 12 (D12) (with values of 0.117 versus 0.017), lower indices were observed for piglets belonging to sows fed with βG-(1,3) (*p* < 0.05).

## 4. Discussion

The results of the indicators of body condition, backfat thickness, and backfat difference (Table 2) between different reproductive moments were not different between treatments, in agreement with Szuba-Trznadel et al. [22], who worked with three concentrations of βG-(1,3)-(1,6) in diets for gestating and lactating sows versus a control group and did not observe any differences between treatments for body weight loss during lactation. However, the results are contrary to those obtained by Szuba-Trznadel et al. [21], who observed less body weight loss when sows were supplemented with βG-(1,3)-(1,6) and βG-(1,3)-(1,6) and mannan.

The similar body condition of sows between treatments is supported by the absence of difference we observed in feed intake and feed conversion variables. Our results correspond to those obtained by Szuba-Trznadel et al. [21], who did not observe an increase in feed intake for lactating females supplemented with three different doses of βG-(1,3)-(1,6), but contrast the findings of Chung Wen et al. [25], who observed that sows fed a diet supplemented with 0.4% βG increased feed intake and had less backfat loss during lactation. It should be considered that there are differences in responses between this class of prebiotics, which may be associated with the structure of their molecules and their concentrations in the diets. Furthermore, Kornegay et al. [26] reported in weaned piglets that the promoting effect of βG from yeast also depends on the ingredients that make up the feed, which is a finding that may also apply to sow diets.

For reproductive variables related to the number of born alive, stillborn, mummified, birth weight, birth weight homogeneity, and farrowing efficiency (Table 3), βG-(1,3) did not provide any advantage over the control group (*p* > 0.05), confirming that our findings are in line with other studies [20,22].

However, βG-(1,3) derived from *Euglena gracilis* supported greater colostrum production, which is a result similar to that observed with the use of 2 g of yeast derivatives (mannan oligosaccharides, glucomannoproteins, and βG) per kilogram of feed during gestation in multiparous sows, which showed a 24% increase in colostrum production compared to the control group [27]; these are values similar to those observed in our study (24.9%). These results confirm the potential of this class of prebiotics in promoting immune responses [7,18].

Decaluwé et al. [28] state that sow body condition has a direct influence on colostrum production (Table 3); however, these factors were not different between the evaluated groups (*p* > 0.05). Additionally, colostrum production, as well as its composition, are influenced by external factors such as environmental stress and by those inherent to the sow such as nutritional status [29], circulating hormones, immune status, characteristics of the litter itself, especially birth vitality [30], and total litter weight [31]. The interactions between these factors are complex and make it difficult to elucidate their respective influences on colostrogenesis. Although in our study no effect was observed on the number of live born and birth weight, sows fed diets supplemented with βG-(1,3) produced more colostrum than the control group.

Beta-glucan (BG) is a well-known substance with immunomodulatory properties that is capable of stimulating the immune system. The underlying mechanism by which BG-(1,3) enhances antibody production involves the activation of key immune cells, including macrophages, monocytes, and dendritic cells. Binding to specific receptors on the surfaces of these cells, βG-(1,3) triggers a cascade of immune responses [8,17]. Activation of immune cells by βG-(1,3) leads to the release of pro-inflammatory cytokines, which subsequently promote the production and maturation of antibody-secreting B cells. Upon activation, B cells produce and release antigen-specific antibodies (IgG, IgA, and IgM), thereby providing a crucial defense mechanism against invading pathogens such as bacteria or viruses [8,20]. Consequently, βG-(1,3) indirectly augments antibody production by modulating immune cell activity and facilitating a more robust immune response via colostrum.

The greater colostrum production observed determined a higher intake of colostrum by piglets during the first 18 h of life (Table 3), which is a condition that has determining effects on their health [23,29,32,33]. In this perspective, piglets consuming 20% of their birth weight in colostrum are satisfactorily supplied with energy and nutrients to increase their body mass and maintain homeothermy [34].

Newborn piglets consuming 10% of their birth weight in colostrum satisfy basic maintenance needs for survival, but body temperature is compromised and weight gain in 24 h becomes minimal [34], which may explain the superior weight gain in 50% of the piglets belonging to the βG-(1,3) group at 18 h post birth (*p* < 0.05). Piglets belonging to the βG-(1,3) group weighed 1337 g at birth and consumed 292 g of colostrum (21.84% of body weight), whereas piglets from the control group weighed 1342 g at birth and consumed 234 g of colostrum (17.43% of body weight). This difference of 4.41% in colostrum intake was reflected in the weight gain of piglets belonging to the βG-(1,3) group at 18 h post birth.

In this study, it was observed that the body weight gain of piglets increases concomitantly with colostrum intake during the first 24 h after birth. An average weight gain of 50 g was found to be a result of colostrum consumption of approximately 250 g [23]. Piglets born from sows fed with βG-(1,3) consumed on average 294 g and gained 102 g, whereas control piglets consumed an average of 234 g and gained 68 g.

It is recognized that not only the production of colostrum impacts piglet health and performance, but also its nutritional composition. Colostrum is rich in proteins, carbohydrates, and lipids, as well as minerals, vitamins, leukocytes, and somatic cells in lower concentrations [35,36], and contains additional bioactive compounds, including components of the sow’s immune system [37,38]. The proportions of these components can be modulated by different factors, including additives such as the one evaluated in this study [39].

The higher values of colostrum concentrations of IgG and IgM and of IgG, IgA, and IgM in piglets’ plasma (Table 4), in favor of the group treated with βG-(1,3), correspond to those found in the literature [25,40]. Immunoglobulins in colostrum are derived from maternal blood, and their blood concentrations at the end of gestation explain 36% of the observed variability in colostrum concentrations at parturition [41]. This statement supports the predicted immunomodulatory action of βG-(1,3) [7,10,11,14], which was administered to the feed of sows starting at day 85 of gestation, and is in line with a large number of studies that regularly evaluate dietary supplementation with various ingredients (e.g., fish oil, fermented liquid feed, mannanoligosaccharides) that presumably have immunomodulatory effects on colostrum immunoglobulin content or piglet immune status [29,31,35,41,42].

The immune responses triggered by βG result from their action as modifiers of biological response and their recognition as pathogen-associated molecular patterns by innate surface receptors of the intestine [8]. This plateau may have been reached first by piglets belonging to the βG-(1,3) group due to their consumption of more than 250 g of colostrum. Increasing colostrum production and intake (Table 3) is an objective in favor of improving piglet immunological protection through a greater supply of antibodies (Table 4), representing an important action in the current context of reducing the use of antibiotics in pig production [43]. Piglets with better immunological protection (plasma IgG) from colostrum (with intake above 290 g) showed better performance compared to those consuming a lower amount (<290) until weaning at 28 days of age [42].

The immune responses triggered by βG arise from their role as modifiers of the biological response and their recognition as pathogen-associated molecular patterns by the surface receptors of the individual’s innate immune cells, since these compounds are not synthesized by animals [17,20]. Thus, they are recognized and subsequently phagocytosed and processed by macrophages and dendritic cells present in the intestine. Later, they are conducted via the lymphatic system to different immune organs, such as the spleen, where they are released, conferring to immune cells the initiation of more efficient antimicrobial and inflammatory responses against pathogenic challenges [8]. This mode of action is consistent with the results obtained, in which, as mentioned, the treated group showed the highest concentrations of IgG, IgA, and IgM (Table 4).

Several studies have reported a significant increase in colostral IgG, IgA, and/or IgM concentrations, but only two studies have reported positive effects on plasma IgG concentrations [44,45]. Our study demonstrated that dietary supplementation of sows with βG-(1,3) derived from *E. gracilis* promoted a greater production of IgG and IgM, subsequently transferred via colostrum to their piglets (Table 4). It is reiterated that the concentration of Igs in piglet plasma is positively related to colostrum intake (Table 3) and to the concentration of Igs in colostrum (Table 4). In a previous study conducted by Devillers et al. [42], it was observed that plasma IgG concentrations in piglets tended to plateau as colostrum intake exceeded 200–250 g [29]. This observation is attributed to the saturation of intact immunoglobulin absorption. In our study, we did not observe a significant difference in zootechnical performance between the βG-(1,3) supplemented group and the control group until weaning at 21 days of age. The average colostrum intake in both groups was 234 g in the supplemented group and 5.735 g in the control group, which suggests that the plateau effect might not be influenced by βG-(1,3) supplementation (5.892 vs. 5.735). Although differences in colostral consumption and quality favored piglets from sows treated with βG-(1,3), a minimum consumption of 200 g of colostrum per piglet influences cell proliferation and intestinal development, as well as determining significant changes in the shape, size, and density of villi [42,46]; this amount of colostrum intake may be sufficient to not compromise performance, which was the result we observed when comparing piglet performance in both groups until weaning (Table 5).

The weight of piglets at different ages until weaning (Table 5) remained similar between the control and βG-(1,3) treatment groups, corresponding to the same result obtained by Szuba-Trznadel et al. [21], who supplemented sow diets with βG-(1,3)-(1,6) and βG-(1,3)-(1,6) plus mannan and weaned their offspring at 22 days of age.

The pre-weaning diarrhea index, considering an evaluation by periods (Table 6), did not determine any advantage for piglets born and nursed by sows that received βG-(1,3). However, there was a difference (*p* < 0.05) in favor of the treated group when daily evaluations of diarrhea were considered, with lower rates on days 8 and 12 of lactation.

Diarrhea in lactating piglets is multifactorial and may involve bacterial, viral, and parasitic agents [47], which does not always confer complete control of these conditions to an additive. However, the indications of improvement in the number of diarrhea episodes can be supported considering the actions that βG determines. The use of 108 ppm of βG extracted from *Euglena gracilis* in the feed of piglets infected with *E. coli* stimulated T cell activation and reduced intestinal inflammation and diarrhea episodes, additionally improving intestinal barrier function [7]. This immune mediation with health repercussions was also observed in a study that compared various sources of βG (*Laminaria digitata, Sclerotium rolfsii*, *Alcaligenes faecalis, Saccharomyces cerevisiae*, and *Euglena gracilis*), with *Euglena gracilis* identified as the most effective in stimulating the immune response, acting on the increase in lymphocyte proliferation and cytokine production [17].

## 5. Conclusions

Supplementing sows with βG-(1,3) derived from *Euglena gracilis*, starting from late gestation, increased both the quantity and quality of colostrum produced in terms of both quantity and immunity (IgG, IgA, and IgM), respectively. These findings suggest that enhancing immunity through βG-(1,3) supplementation may serve as a valuable tool in modern pig farming, aligning with the industry’s goal of reducing the use of growth-promoting antibiotics.

## Figures and Tables

**Table 1:** Composition and estimated nutritional and energetic values of experimental diets for sows in gestation and lactation with βG-(1,3).

Ingredients (%)	Phases	
	Gestation	Lactation
Corn 7.5%	54.39	51.60
Soybean meal 45%	26.50	27.00
Cookie meal	10.00	9.00
Meat and bone meal 44%	3.92	5.00
Soybean oil	1.36	2.55
Soybean hulls	1.25	2.00
Premix for sows	1.50 ^1^	1.50 ^1^
Salt	0.30	0.40
Limestone 37%	0.20	0.33
L-carnitine	0.20	0.20
Sodium bicarbonate 27%	0.25	0.10
L-lysine 80%	0.00	0.07
L-threonine 98%	0.00	0.06
DL-methionine 98%	0.00	0.04
Palatability enhancer	0.00	0.02
Vitamin D_3_	0.01	0.01
Mycotoxin binder	0.10	0.10
Beta-glucans ^●^	0.02	0.02
Total	100	100
Nutrients estimated		
Metabolizable energy—(kcal/kg)	3325	3350
Crude protein (%)	18.500	19.136
Lysine digestible (%)	1.044	1.127
Methionine + cysteine digestible (%)	0.854	0.897
Threonine digestible (%)	0.726	0.797
Tryptophan digestible (%)	0.466	0.469
Valine digestible (%)	0.829	0.848
Crude fiber (%)	3.653	3.888
Total fat (%)	5.262	6.349
Total calcium (%)	0.700	0.900
Total phosphorus (%)	0.586	0.648
Available phosphorus (%)	0.400	0.460
Sodium (%)	0.320	0.322
Dry matter (%)	87.264	87.564

^●^ ALETA^TM^ (dried *Euglena gracilis* algae). ^1^ Values per kg of product: Copper, 10,000,000 mg/kg; Iron, 100,000,000 mg/kg; Manganese, 40,000,000 mg/kg; Cobalt, 1,000,000 mg/kg; Iodine, 1,500,000 mg/kg; Zinc, 100,000,000 mg/kg, vitamin A, 10,000.00 IU/g; vitamin D3, 2000.00 IU/g; vitamin E, 50,000.00 IU/kg; Vitamin K3, 2000.00 mg/kg; Vitamin B1 (Thiamine), 2000.00 mg/kg; Vitamin B2 (Riboflavin), 6000.00 mg/kg; Vitamin B6 (Pyridoxine), 3000.00 mg/kg; Vitamin B12 (Cyanocobalamin), 30,000.00 mcg/kg; Calcium Pantothenate, 10,000.00 mg/kg; Biotin, 200.00 mg/kg; Folic Acid, 3000.00 mg/kg; Niacin, 30,000.00 mg/kg; Selenium, 300.00 mg/kg; Ethoxyquin, 52.083 mg/kg; BHA, 41.667 mg/kg.

**Table 2:** Backfat thickness (BT) and feed conversion rate (FCR) of sows fed with beta-glucans and control diet on the day during the gestation and lactation phase.

Variables	Control	*β*-Glucans (1,3)	CV (%)	*p*-Value
Backfat thickness D85 (mm)	12.32	11.88	22.01	0.1866
Backfat thickness D0 (mm)	14.38	14.03	18.28	0.5557
Backfat thickness D21 (mm)	12.81	12.25	19.69	0.2345
Difference BT D85 to D0 (mm)	2.06	2.15	91.7	0.2487
Difference BT D1 to D21 (mm)	−1.57	−1.78	112.9	0.1698
FCR * up to D10	2.142	2.122	36.7	0.9213
FCR * up to D18	2.026	2.134	29.1	0.4880
Average feed intake D21	6.49	6.40	20.7	0.7789

CV = coefficient of variation. D85 = 85th day of gestation, D0 = day of parturition, D10 = 10th day of lactation, D18 = 18th day of lactation and D21 = weaning day. * FCR = total sow feed consumption in the period/total piglet weigh gain in the period. A total of 60 sows were assigned to each treatment group.

**Table 3:** Reproductive performance, parturition interventions, piglet metrics, serum glucose, and colostrum variables of sows fed with beta-glucan and control diet.

Variables	Control	*β*-Glucans (1,3)	CV (%)	*p*-Value
Total born (n)	16.79	16.31	23.0	0.2931
Live born (n)	15.34	14.79	24.3	0.3590
Stillborn (n)	0.92	0.90	144.8	0.5123
Mummification (n)	0.55	0.45	187.5	0.0948
Average weight at birth (kg)	1.342	1.337	15.3	0.9263
Piglets below 900 g (n)	1.76	1.56	113.9	0.6832
Piglets below 900 g (%)	10.07	10.24	109.7	0.9423
Dystocia (n)	3	1	-	0.5371
Dystocia (%)	5.00	1.66		-
Oxytocin (n)	7	3	-	0.3153
Oxytocin (%)	11.66	5.00	-	-
Time of delivery (min)	224.15	255.74	41.6	0.1677
Weight at 18 h (kg) *	1.403	1.426	14.9	0.7316
Serum glucose of sow at 0 h post-partum (mg/dL) *	71.370	74.533	14.2	0.2566
Serum glucose of sow at 2 h post-partum (mg/dL) *	73.778	76.233	18.8	0.5198
Serum glucose of sow at the end of parturition (mg/dL) *	77.120	77.068	28.2	0.9932
Colostrum production up to 18 h (kg) *	3.476 ^b^	4.343 ^a^	41.89	0.0450
Colostrum intake up to 18 h (g) *	234.92 ^b^	294.05 ^a^	40.1	0.0394
Piglet weight gain up to 18 h (g) *	68 ^b^	102 ^a^	69.3	0.0354
Colostrum intake by piglet weight (%)	17.4 ^b^	21.8 ^a^	49.8	0.0000

^a,b^ Different letters in the rows indicate a significant difference by Student’s *t*-test and Wilcoxon–Mann–Whitney test (*p* < 0.05). * n = 30 sows per treatment. Variables without asterisks (*) were based on data from 60 sows per treatment.

**Table 4:** Means of IgG, A, and M values in colostrum and serum of piglet at D4 of age from sows fed with β-Glucans (1,3) and control diet.

Colostrum	Treatments		CV (%)	*p*-Value
	Control	*β*-Glucans (1,3)		
IgG (mg/mL)	36.594 ^b^	42.508 ^a^	10.9	0.0000
IgA (mg/mL)	6.462	10.332	72.9	0.0699
IgM (mg/mL)	2.320 ^b^	2.698 ^a^	18.3	0.0161
Serum				
IgG (mg/mL)	27.038 ^b^	40.894 ^a^	41.1	0.0033
IgA (mg/mL)	14.278 ^b^	31.439 ^a^	88.5	0.0236
IgM (mg/mL)	9.135 ^b^	22.361 ^a^	112.2	0.0270

^a,b^ Different letters in the rows indicate a significant difference by Wilcoxon–Mann–Whitney test (*p* < 0.05); trend (*p* < 0.10). CV = coefficient of variation. A total of 20 sows were assigned to each treatment group.

**Table 5:** Piglet count, piglets weight, litter weight, daily piglet weight gain (DWG), and piglet mortality from sows fed beta-glucans and control diet on days 2 (D2), 10 (D10), 18 (D18), and 21 (D21) of age.

Variables	Control	*β*-Glucans (1,3)	CV (%)	*p*-Value
Piglets at D2 (n)	14.98	14.98	4.5	0.7326
Piglets weight at D2 (kg)	1.47	1.48	25.2	0.9425
Litter weight at D2 (kg)	21.87	22.05	23.7	0.8810
Piglets at D10 (n)	14.67	14.60	4.9	0.6767
DWG to D10 (kg)	0.20	0.21	26.0	0.5745
Piglets weight at D10 (kg)	3.06	3.16	21.7	0.6897
Litter weight at D10 (kg)	43.26	45.92	25.4	0.7994
Piglets at D18 (n)	14.49	14.28	5.4	0.2355
DWG to D18 (kg)	0.23	0.23	20.3	0.6119
Piglets weight at D18 (kg)	5.06	5.20	19.0	0.6794
Litter weight at D18 (kg)	73.32	74.06	19.2	0.9867
Piglets at D21 (n)	14.49	14.28	5.4	0.2355
DWG to D21 (kg)	0.23	0.23	20.3	0.6119
Piglets weight at D21 (kg)	5.74	5.89	19.1	0.6686
Litter weight at D21 (kg)	83.09	84.00	19.3	0.9773
Piglets’ mortality (%)	3.21	4.60	125.5	0.1285

CV = coefficient of variation. Piglets were derived from a pool of 60 sows per treatment group.

**Table 6:** Number of piglets with diarrhea, diarrhea index, and total number of litters with diarrhea (TLD) on days 2 (D2), 10 (D10), 18 (D18), and 21 (D21) of age according to experimental treatments.

Variables	Control	*β*-Glucans (1,3)	*p*-Value
Diarrhea D2–D10 (n)	76	62	0.2094
Diarrhea D2–D10 (%)	8.64	7.06	-
Diarrhea index D2–D10	0.085	0.069	-
Diarrhea D11–D18 (n)	37	42	0.5578
Diarrhea D11–D18 (%)	4.24	4.87	-
Diarrhea index D11–D18	0.042	0.047	-
Diarrhea D19–D21 (n)	3	3	1.0000
Diarrhea D19–D21 (%)	0.34	0.34	-
Diarrhea index D19–D21	0.003	0.003	-
Diarrhea D2–D21 (n)	116	107	0.5080
Diarrhea D2–D21 (%)	12.94	11.91	-
Diarrhea index D2–D21	0.129	0.124	-
TLD * (n)	18	12	0.2091

CV = coefficient of variation. * Considered the litter with more than 20% of piglets with diarrhea. Piglets were derived from a pool of 60 sows per treatment group.

## Data Availability

The datasets generated and/or analyzed during the current study are available from the corresponding author on reasonable request.
